# Supplementary material for: Chest computed tomography outcomes in a randomized clinical trial in cystic fibrosis: Lessons learned from the first ataluren phase 3 study
Source: PLoS One. 2020 Nov 3;15(11):e0240898. doi: 10.1371/journal.pone.0240898 (PMC7608929; doi:10.1371/journal.pone.0240898)
Supplement: S1 File — (DOCX) [file pone.0240898.s001.docx]

**S1 File**

**Title: Chest CT outcomes in a phase 3 clinical trial in cystic fibrosis: Lessons learned from the first ataluren study**

**Contents**

**1. Methods**

1.1 Inclusion and exclusion criteria first PTC phase III study

1.2 Training of observers to score CTs using PRAGMA-CF

1.3 Power analysis

**2. Tables**

S1 Table 1: CT scanner parameter settings

S1 Table 2: Intra-ICC scores

S1 Table 3: CF-CT and PRAGMA-CF subscores for the ataluren and placebo group

S1 Table 4: Linear mixed effects models

S1 Table 5: Power analysis

**3 S Figures**

S1 Fig 1A: Bland Altman plots for %Bronchiectasis

S1 Fig 1B: Bland Altman plots for %Disease

S1 Fig 1C: Bland Altman plots for %Mucus Plugging

S1 Fig 2A: Regression Model Bronchiectasis PRAGMA-CF versus CF-CT SOS

S1 Fig 2B: Regression Model Bronchiectasis PRAGMA-CF versus CF-CT EOS

S1 Fig 3A: Regression Model Mucus Plugging PRAGMA-CF versus CF-CT SOS

S1 Fig 3B: Regression Model Mucus Plugging PRAGMA-CF versus CF-CT EOS

**1. Methods**

**1.1 Inclusion and exclusion criteria first PTC phase III study**

Most important inclusion criteria included age ≥6 years; abnormal nasal potential difference; sweat chloride >40 mmol/L; documentation of the presence of a nonsense mutation in at least one allele of the CFTR gene ; the ability to perform a valid, reproducible spirometry test that demonstrated an FEV_1_≥40% and ≤90% of predicted for age, gender, and height; confirmed screening laboratory values within pre-specified central laboratory ranges; and willingness and ability to comply with all study procedures, scheduled visits, and restrictions.

Major exclusion criteria included known hypersensitivity to ataluren; any change in a chronic treatment or prophylaxis regimen for CF within four weeks of starting study treatment; systemic aminoglycoside antibiotics treatment within two weeks before the date of baseline nasal potential difference assessment; major complications of lung disease within eight weeks prior to start of study; history of grade 3 or higher creatinine elevation due to aminoglycoside nephrotoxicity; and ongoing participation in any other clinical trial. For additional details on the study population and study details we refer to the publication by Kerem et al [1]

**1.2 training of observers**

Observers are trained according to the LungAnalysis standard operating procedure. This includes firstly to go through the CF-CT training module which consists of a theoretical part and a practical part. Each observer has to score 7 training sets according to the scoring instructions. After completion of each test set the inter observer correlation (ICC) coefficient is computed by comparing the results of the observer with those of the mean gold standard scoring results by 2 independent experienced radiologist observers. The results are being discussed with the head of the LungAnalysis laboratory, focusing on CT scans with clear discrepancies in scores. Once ICC are comparable to those of the gold standard the observer goes through a similar training procedure for PRAGMA-CF

**1.3 power analysis**

Using the data and the mixed-effects models fitted from the PTC study we performed a simulation analysis to obtain the power when assuming that a 10, 30 and 50% reduction from SOS to EOS will be obtained when treatment is initiated. In particular, we assume that %Disease PRAGMA-CF at SOS will be 9.94 and %Disease PRAGMA-CF at EOS will be -9·94x0·1, -9·94x0·3 and -9·94x0·5.  The variance of the outcome is in that case 10.5. %MP PRAGMA-CF at SOS will be 2·14 and %MP PRAGMA-CF at EOS will be -2·14x0·1, -2·14x0·3 and -2·14x0·5. The variance of the outcome is in that case 2.1.

**2 S Tables**

**S Table 1: CT scan parameter settings**

This table shows the CT scanner parameter settings used for acquisition of 408 chest CT scans at start (SOS) and end of treatment (EOS). Of 12 CT scans the CT scanner manufacturer and type was unknown.

| **CT scanner Manufacturer** | **CT scanner Type** | **CT scans (n)** | **Average KVP** | **Most used KVP** | **Reconstruction Kernel** | **Most used Reconstruction Kernel** | **Average FOV** | **Average Thickness** | **Most used Thickness** |
| --- | --- | --- | --- | --- | --- | --- | --- | --- | --- |
| GE medical systems | Discovery CT750 HD | 4 | 116 | 120 | Lung, Bone | Lung | 331 | 1·19 | 1·25 |
|  | LightSpeed Pro 32 | 10 | 120 | 120 | Bone | Bone | 293 | 1·20 | 1·25 |
|  | LightSpeed VCT | 93 | 119 | 120 | Lung, Bone | Bone | 329·82 | 1·24 | 1·25 |
|  | LightSpeed16 | 49 | 117 | 120 | Bone, Standard | Bone | 384·44 | 1·25 | 1·25 |
|  | Optima CT660 | 21 | 120 | 120 | Bone | Bone | 328·34 | 1·24 | 1·25 |
| Philips | Brilliance 16P | 12 | 115 | 120 | L, B | L | 287·54 | 1·08 | 1·00 |
|  | Brilliance 64 | 35 | 120 | 120 | L, B | L | 313·7 | 1·14 | 1·00 |
|  | iCT 256 | 12 | 120 | 120 | L | L | 336·5 | 1·23 | 1·25 |
| Siemens | Definition | 28 | 120 | 120 | B35s, B60f, B60s, B70s | B60s, B60f | 344·7 | 1·00 | 1·00 |
|  | Sensation 16 | 38 | 118 | 120 | B60s, B60f | B60f | 378·9 | 1·07 | 1·00 |
|  | Sensation 40 | 12 | 120 | 120 | B60s, B60f | B60s, B60f | 305·9 | 1·00 | 1·00 |
|  | Sensation 64 | 55 | 120 | 120 | B60s, B60f | B60s, B60f | 305·9 | 1·00 | 1·00 |
|  | Sensation Cardiac 64 | 12 | 120 | 120 | B60s, B60f | B60s, B60f | 345·7 | 1·07 | 1·00 |
|  | Somatom Definition | 2 | 120 | 120 | B60s, B60f | B60s | 329 | 1·07 | 1·00 |
|  | Volume Zoom | 2 | 120 | 120 | B60f, B70f | B60f, B70f | 282·5 | 1·25 | 1·00 |
| Toshiba | Aquilion | 23 | 120 | 120 | FC53, FC12 | FC53 | 347·7 | 1·08 | 1·00 |

**S Table 2**: Intra-ICC scores for the CF-CT scoring method and PRAGMA-CF scoring method.

| **CF-CT scoring method** | |
| --- | --- |
| Bronchiectasis | 0∙84 |
| Airway Wall Thickening | 0∙98 |
| Mucus Plugging | 0∙92 |
| Atelectasis/consolidations | 0∙73 |
| % Disease | 0.99 |
| **PRAGMA-CF scoring method** | |
| Bronchiectasis | 0∙91 |
| Airway Wall Thickening | 0∙68 |
| Mucus Plugging | n.a.* |
| Atelectasis/consolidations | 0∙10 |
| % Disease | 0∙93 |

*: could not be computed because to many 0 values.

**S Table 3A:** The intention to treat (ITT) population for the ataluren patients.

| **Method** | **Subscore** | **n** | **SOS-CT** | **n** | **EOS-CT** | **Δ** |
| --- | --- | --- | --- | --- | --- | --- |
| CF-CT | %Bronchiectasis | 97 | 14·79 (8·45) | 99 | 15·64 (9·3) | 0·85 |
| CF-CT | %AWT | 97 | 17·7 (9·09) | 99 | 17·77 (8·85) | 0·07 |
| CF-CT | %Mucus Plugging | 97 | 28·35 (9·75) | 99 | 28·70 (11·27) | 0·35 |
| CF-CT | %Disease | 97 | 17·65 (6·58) | 99 | 18·14 (6·93) | 0·49 |
| CF-CT | %Trapped Air | 97 | 14·26 (5·87) | 99 | 14·81 (4·94) | 0·55 |
|  | | | | | | |
| PRAGMA-CF | %Bronchiectasis | 97 | 7·77 (6·49) | 99 | 8·79 (7·08) | 1·02 |
| PRAGMA-CF | %AWT | 97 | 0·02 (0·09) | 99 | 0·02 (0·07) | 0·00 |
| PRAGMA-CF | %Mucus Plugging | 97 | 2·02 (2·23) | 99 | 2·47 (2·54) | 0·45 |
| PRAGMA-CF | %Disease | 97 | 9·82 (7·45) | 99 | 11·30 (8·21) | 1·48 |
| PRAGMA-CF | %Trapped Air | 97 | 0·12 (0·17) | 99 | 0·11 (0·19) | -0·01 |

**S Table 3B:** The intention to treat (ITT) population for the placebo patients**.**

| **Method** | **Subscore** | **n** | **SOS-CT** | **n** | **EOS-CT** | **Δ** |
| --- | --- | --- | --- | --- | --- | --- |
| CF-CT | %Bronchiectasis | 98 | 15·26 (9·65) | 97 | 16·95 (10·05) | 1·69 |
| CF-CT | %AWT | 98 | 17·91 (9·72) | 97 | 19·07 (8·90) | 1·16 |
| CF-CT | %Mucus Plugging | 98 | 29·73 (11·52) | 97 | 29·9 (11·25) | 0·17 |
| CF-CT | %Disease | 98 | 18·22 (6·86) | 97 | 19·22 (7·12) | 1·00 |
| CF-CT | %Trapped Air | 98 | 14·81 (5·32) | 97 | 15·27 (5·79) | 0·46 |
|  | | | | | | |
| PRAGMA-CF | %Bronchiectasis | 98 | 7·82 (6·66) | 97 | 8·92 (7·90) | 1·10 |
| PRAGMA-CF | %AWT | 98 | 0·01 (0·03) | 97 | 0·01 (0·07) | 0·00 |
| PRAGMA-CF | %Mucus Plugging | 98 | 2·26 (2·69) | 97 | 2·14 (2·20) | -0·12 |
| PRAGMA-CF | %Disease | 98 | 10·1 (7·73) | 97 | 11·09 (8·73) | 0·99 |
| PRAGMA-CF | %Trapped Air | 98 | 0·12 (0·18) | 97 | 0·15 (0·32) | 0·03 |

**S Table 4: Linear mixed effects models outcomes**

**S Table 4A**

In this table the linear mixed effects models are shown for the outcomes: CF-CT %Bronchiectasis; CF-CT %AWT; CF-CT %Mucus Plugging; CF-CT %Disease. Confounders included in the models were: Time: SOS/EOS, Tobramycin treatment: yes/no and Treatment group: ataluren/Placebo.

| **CF-CT %Bronchiectasis** | | | |
| --- | --- | --- | --- |
|  | **Value** | **Standard Error** | **p-value** |
| (Intercept) | 15·093 | 1·120 | <0·0001 |
| patient CT status (EOS) | -0·011 | 0·645 | 0·9870 |
| Treatment group (placebo) | 0·296 | 1·468 | 0·8404 |
| Tobramycin treatment (Yes) | 0·167 | 1·791 | 0·9260 |
| patient CT status (EOS) : Treatment group (placebo) | 1·029 | 0·799 | 0·1996 |
| patient CT status (EOS) : Tobramycin treatment (Yes) | 1·050 | 0·812 | 0·1979 |
| Treatment group (placebo) : Tobramycin treatment (Yes) | -0·889 | 2·458 | 0·7180 |
| **CF-CT %AWT** | | | |
| (Intercept) | 17·520 | 1·112 | <0·0001 |
| Patient CT status (EOS) | 0·005 | 0·755 | 0·9948 |
| Treatment group (placebo) | 0·454 | 1·484 | 0·7602 |
| Tobramycin treatment (Yes) | 1·308 | 1·762 | 0·4590 |
| Patient CT status (EOS) : Treatment group (placebo) | 0·985 | 0·936 | 0·2942 |
| Patient CT status (EOS) : Tobramycin treatment (Yes) | -0·380 | 0·952 | 0·6905 |
| Treatment group (placebo) : Tobramycin treatment (Yes) | -1·794 | 2·388 | 0·4534 |
| **CF-CT %Mucus Plugging** | | | |
| (Intercept) | 28·425 | 1·348 | <0·0001 |
| patient CT status (EOS) | 0·287 | 0·896 | 0·7494 |
| Treatment group (placebo) | 0·683 | 1·795 | 0·7041 |
| Tobramycin treatment (Yes) | -0·668 | 2·139 | 0·7551 |
| patient CT status (EOS) : Treatment group (placebo) | -0·436 | 1·110 | 0·6950 |
| patient CT status (EOS) : Tobramycin treatment (Yes) | 0·142 | 1·129 | 0·9002 |
| Treatment group (placebo) : Tobramycin treatment (Yes) | 3·804 | 2·904 | 0·1916 |
| **CF-CT %Diseas**e | | | |
| (Intercept) | 17·898 | 0·816 | <0·0001 |
| patient CT status (EOS) | 0·168 | 0·439 | 0·7020 |
| Treatment group (placebo) | -0·011 | 1·059 | 0·9921 |
| Tobramycin treatment (Yes) | 0·084 | 1·309 | 0·9488 |
| patient CT status (EOS) : Treatment group (placebo) | 0·461 | 0·544 | 0·3976 |
| patient CT status (EOS) : Tobramycin treatment (Yes) | 0·263 | 0·553 | 0·6348 |
| Treatment group (placebo) : Tobramycin treatment (Yes) | 0·667 | 1·805 | 0·7120 |

**S Table 4B**

In this table linear mixed effects models are shown for the outcomes: PRAGMA-CF %Bronchiectasis; PRAGMA-CF %Mucus Plugging; PRAGMA-CF %AWT; PRAGMA-CF %Disease; FEV_1_ %predicted. Confounders included in the models were: Time: SOS/EOS, Tobramycin treatment: yes/no and Treatment group: ataluren/Placebo.

| **PRAGMA-CF %Bronchiectasis** | | | |
| --- | --- | --- | --- |
|  | **Value** | **Standard Error** | **p-value** |
| (Intercept) | 2·523 | 0·156 | <0·0001 |
| patient CT status (EOS) | 0·139 | 0·079 | 0·0795 |
| Treatment group (placebo) | -0·149 | 0·200 | 0·4568 |
| Tobramycin treatment (Yes) | -0·061 | 0·250 | 0·8065 |
| patient CT status (EOS) : Treatment group (placebo) | -0·011 | 0·098 | 0·9109 |
| patient CT status (EOS) : Tobramycin treatment (Yes) | 0·083 | 0·099 | 0·4055 |
| Treatment group (placebo) : Tobramycin treatment (Yes) | 0·258 | 0·347 | 0·4570 |
| **The square root PRAGMA-CF %Mucus Plugging** | | | |
| (Intercept) | 1·114 | 0·098 | <0·0001 |
| patient CT status (EOS) | 0·175 | 0·080 | 0·0292 |
| Treatment group (placebo) | 0·124 | 0·132 | 0·3501 |
| Tobramycin treatment (Yes) | 0·226 | 0·154 | 0·1435 |
| patient CT status (EOS) : Treatment group (placebo) | -0·129 | 0·099 | 0·1910 |
| patient CT status (EOS) : Tobramycin treatment (Yes) | -0·116 | 0·100 | 0·2478 |
| Treatment group (placebo) : Tobramycin treatment (Yes) | -0·136 | 0·204 | 0·5048 |
| **Square root PRAGMA-CF %AWT** | | | |
| (Intercept) | 0·235 | 0·032 | <0·0001 |
| patient CT status (EOS) | 0·000 | 0·035 | 0·9918 |
| Treatment group (placebo) | 0·013 | 0·044 | 0·7598 |
| Tobramycin treatment (Yes) | 0·001 | 0·049 | 0·9847 |
| patient CT status (EOS) : Treatment group (placebo) | 0·010 | 0·043 | 0·8127 |
| patient CT status (EOS) : Tobramycin treatment (Yes) | -0·035 | 0·044 | 0·4277 |
| Treatment group (placebo) : Tobramycin treatment (Yes) | 0·011 | 0·062 | 0·8536 |
| **Square root PRAGMA-CF %Disease** | | | |
| (Intercept) | 2·839 | 0·161 | <0·0001 |
| patient CT status (EOS) | 0·212 | 0·079 | 0·0079 |
| Treatment group (placebo) | -0·046 | 0·205 | 0·8240 |
| Tobramycin treatment (Yes) | 0·024 | 0·259 | 0·9251 |
| patient CT status (EOS) : Treatment group (placebo) | -0·075 | 0·098 | 0·4427 |
| patient CT status (EOS) : Tobramycin treatment (Yes) | 0·020 | 0·099 | 0·8375 |
| Treatment group (placebo) : Tobramycin treatment (Yes) | 0·156 | 0·358 | 0·6633 |
| **FEV_1_ %Predicted** | | | |
| (Intercept) | 60·517 | 1·879 | <0·0001 |
| patient CT status (EOS) | -1·505 | 1·071 | 0·1618 |
| Treatment group (placebo) | 0·718 | 2·459 | 0·7705 |
| Tobramycin treatment (Yes) | 3·930 | 3·029 | 0·1962 |
| patient CT status (EOS) : Treatment group (placebo) | -1·138 | 1·330 | 0·3935 |
| patient CT status (EOS) : Tobramycin treatment (Yes) | -0·736 | 1·354 | 0·5872 |
| Treatment group (placebo) : Tobramycin treatment (Yes) | -5·572 | 4·146 | 0·1804 |

**S Table 5:** **Power Analysis**

Using the data and the mixed-effects models fitted from the PTC study we performed a simulation analysis to obtain the power when assuming that a 10, 30 and 50% reduction from SOS to EOS will be obtained when treatment is initiated. In particular, we assume that %Disease PRAGMA-CF at SOS will be 9.944385 and %Disease PRAGMA-CF at EOS will be -9·944385x0·1, -9·944385x0·3 and -9·944385x0·5. The variance of the outcome is in that case 10.5. %MP PRAGMA-CF at SOS will be 2·1445593 and %MP PRAGMA-CF at EOS will be -2·1445593x0·1, -2·1445593x0·3 and -2·1445593x0·5. The variance of the outcome is in that case 2.1

**S Table 5A:**

Outcome PRAGMA-CF %Disease: for 10%, 30% and 50% reduction in progression of the active treatment arm relative to the placebo arm we obtain a power as indicated in the table. Total subjects include the number of subjects needed in the active treatment arm and in the placebo arm.

| **Total subjects**  **10% decrease** | **Power**  **10% decrease** | **Total subjects**  **30% decrease** | **Power**  **30% decrease** | **Total subjects 50% decrease** | **Power**  **50% decrease** |
| --- | --- | --- | --- | --- | --- |
| 1590 | 0.700 | 190 | 0.800 | 70 | 0.805 |
| 1630 | 0.785 | 210 | 0.850 | 90 | 0.855 |
| 1670 | 0.745 | 230 | 0.820 | 110 | 0.890 |
| 1710 | 0.770 | 250 | 0.870 | 130 | 0.955 |
| 1750 | 0.760 | 270 | 0.915 | 150 | 0.955 |

**S Table 5B:**

Outcome PRAGMA-CF %Mucus Plugging: for 10%, 30% and 50% reduction in progression of the active treatment arm relative to the placebo arm we obtain a power as indicated in the table. Total subjects include the number of subjects needed in the active treatment arm and in the placebo arm.

| **Total subjects 10% decrease** | **Power**  **10% decrease** | **Total subjects 30% decrease** | **Power**  **30% decrease** | **Total subjects 50% decrease** | **Power**  **50% decrease** |
| --- | --- | --- | --- | --- | --- |
| 1690 | 0.505 | 310 | 0.785 | 110 | 0.750 |
| 1730 | 0.565 | 330 | 0.705 | 130 | 0.755 |
| 1770 | 0.495 | 350 | 0.765 | 150 | 0.815 |
| 1810 | 0.540 | 370 | 0.795 | 170 | 0.890 |
| 1850 | 0.535 | 390 | 0.875 | 190 | 0.940 |

3 **S Figures**

**S Figure 1A**: Bland Altman plots for %Bronchiectasis (%BE); SOS = start of study; EOS = end of study.


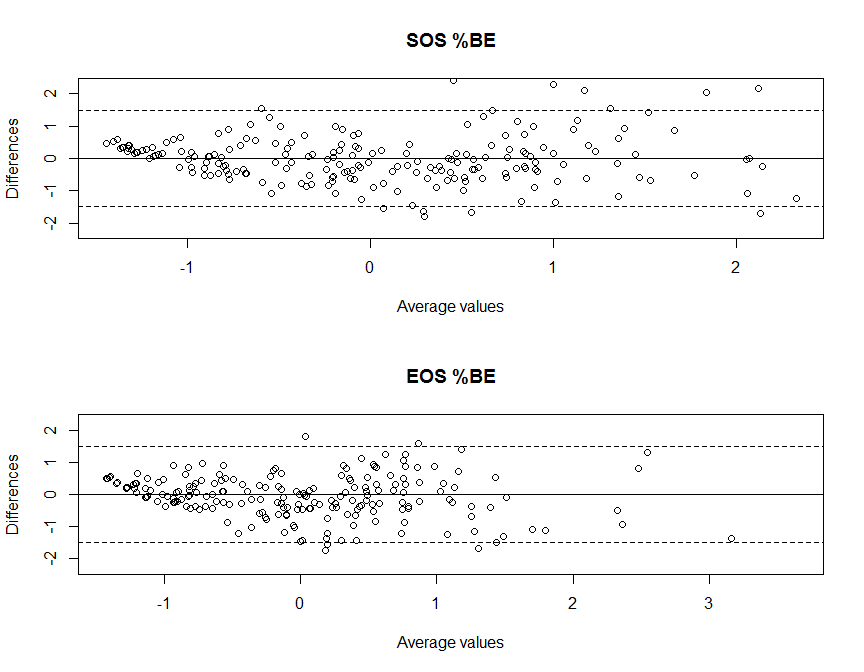


**S Figure 1B**: Bland Altman plots for %Disease. ; SOS = start of study; EOS = end of study.


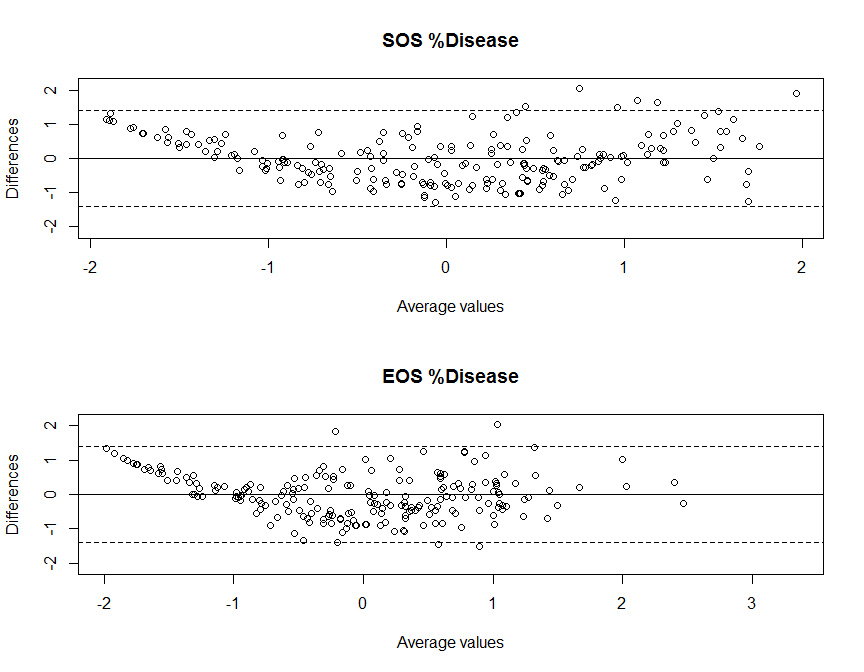


**S Figure 1C:** Bland Altman plots for %Mucus Plugging. ; SOS = start of study; EOS = end of study.

**S**

**Figure**

**1**

Plot of the regression model investigating the association between PRAGMA

-

CF and

CF

-

CT for

%Bronchiectasis (

%

BE) at

start of study (SOS)

(

S

Figure

2

A) and

end of study (

EOS

)

(

S

Figure

2

B). The

black line represents the regression line. The correlation between PRAGMA

-

CF and CF

-

CT for %BE at

SOS and EOS is 0.71.

**S**

**Figure**

**1**

**A**

**S**

**Figure**

**1**

**B**


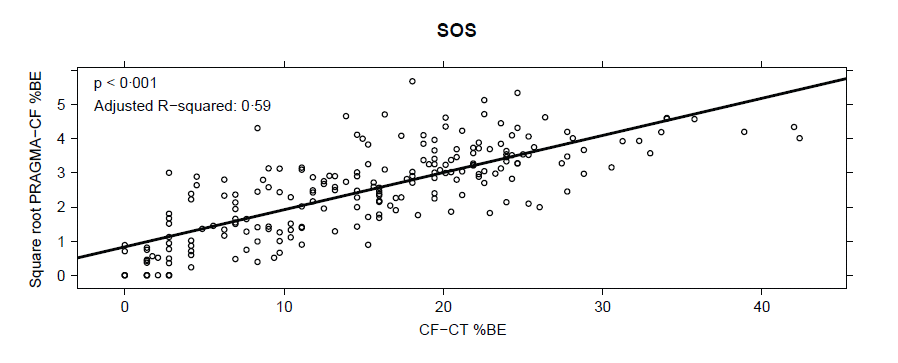

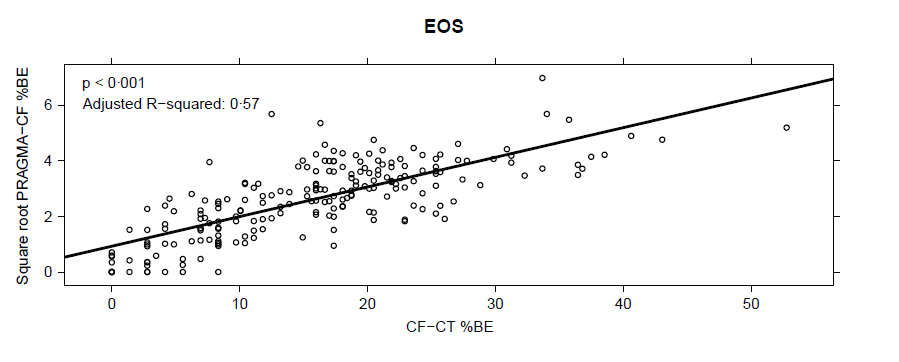


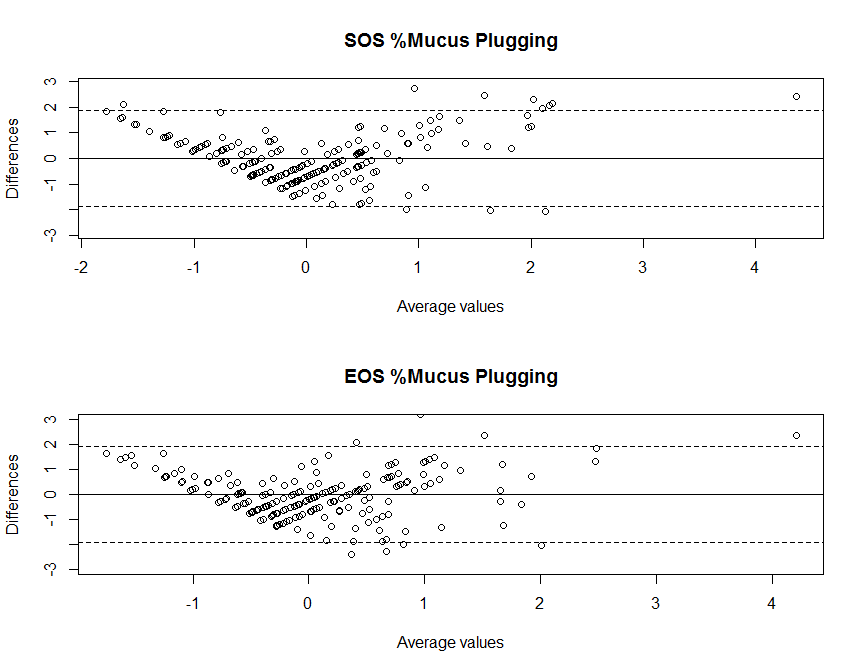


**S Fig 2**

Plot of the regression model investigating the association between PRAGMA-CF and CF-CT for %Bronchiectasis (%BE) at start of study (SOS) (S Figure 2A) and end of study (EOS) (S Figure 2B). The black line represents the regression line. The correlation between PRAGMA-CF and CF-CT for %BE at SOS and EOS is 0.71.

**S Fig 2A**


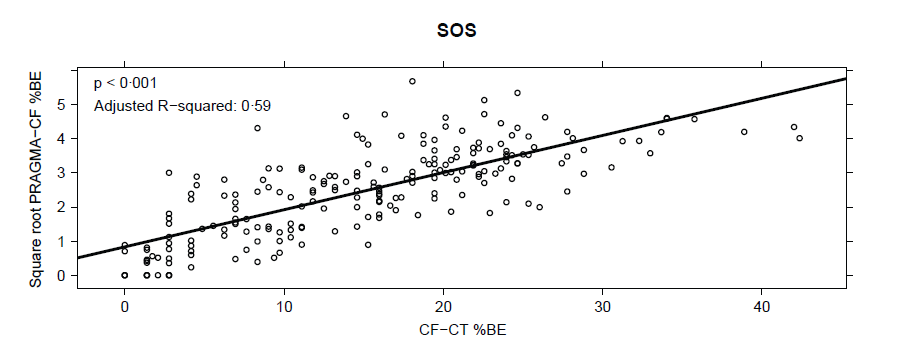


**S Fig 2B**


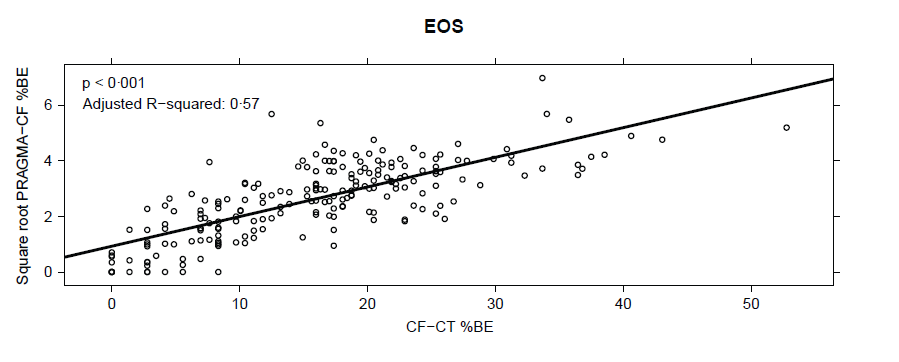


**S Figure 3**

Plot of the regression model investigating the association between PRAGMA-CF and CF-CT for %Mucus Plugging at start of study (SOS) (S Figure 2A) and end of study EOS (S Figure 2B). The black line represents the regression line. The correlation between PRAGMA-CF and CF-CT for %Mucus Plugging at SOS is 0.54 and at EOS is 0.52.

**S Figure 3A**


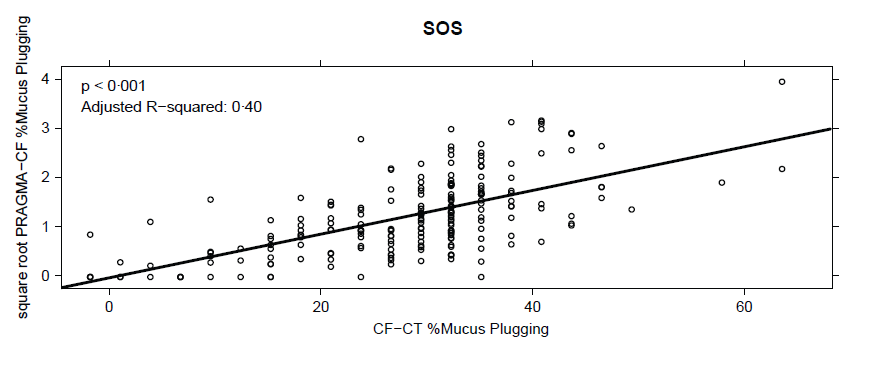


**S Figure 3B**


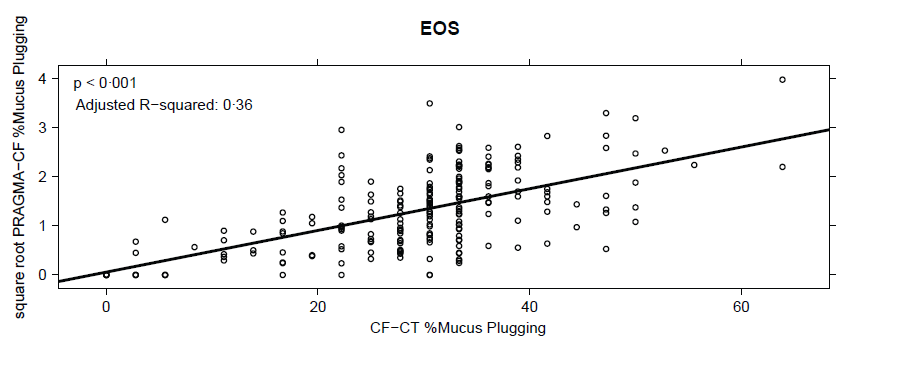

References

1. Kerem E, Konstan MW, De Boeck K, Accurso FJ, Sermet-Gaudelus I, Wilschanski M, Elborn JS, Melotti P, Bronsveld I, Fajac I, Malfroot A, Rosenbluth DB, Walker PA, McColley SA, Knoop C, Quattrucci S, Rietschel E, Zeitlin PL, Barth J, Elfring GL, Welch EM, Branstrom A, Spiegel RJ, Peltz SW, Ajayi T, Rowe SM, Cystic Fibrosis Ataluren Study G. Ataluren for the treatment of nonsense-mutation cystic fibrosis: a randomised, double-blind, placebo-controlled phase 3 trial. *Lancet Respir Med* 2014: 2(7): 539-547.
